# Reducing high dose antipsychotic therapy (HDAT) in a community mental health team (CMHT)

**DOI:** 10.1192/bjo.2021.325

**Published:** 2021-06-18

**Authors:** Richard Walsh, Sonn Patel, Valentina Loddo, Rebecca Fahy, Elizabeth Walsh

**Affiliations:** 1 School of Medicine, University College Dublin; 2 Galway University Hospital; 3 Semmelweis University

## Abstract

**Aims:**

The consensus statement (CR190) of The Royal College of Psychiatrists states that the benefit of prescribing HDAT does not outweigh the risk of the increased side effect burden. HDAT is defined as the “daily dose of a single antipsychotic exceeding the upper limit for that drug as stated in the Summary of Product Characteristic (SPC) or British National Formulary (BNF),” and as the cumulative daily dose of two or more antipsychotics (for combined prescription). The prevalence of HDAT has been shown to vary widely and protocols for monitoring poorly implemented. In 2018 we completed a baseline survey of the prevalence of HDAT within our CMHT. We assessed our prescribing practice as compared to seven best practice audit criteria, which were adopted. Our aim is to resurvey closing the audit loop to 1) establish the current prevalence of HDAT and 2) assess the impact the intervention on prescribing practice.

**Method:**

Multi-disciplinary case notes for all registered patients were studied. A database was created including sociodemographic details, chart diagnosis, and medication. The proportion of patients prescribed antipsychotic medication was identified. The dose of each medication was converted into a percentage of BNF maximum recommended dose for that drug. For combined antipsychotic prescription, the cumulative dose was obtained adding the single percentages together. Exceeding 100% was regarded as HDAT. All HDAT patients were assessed against identified audit criteria as outlined by the Humber NHS Foundation Trust.

**Result:**

Of a total of 246 patients, 177 (72%) were prescribed antipsychotic medication. Of these, 14 (8%) were in receipt of HDAT. This compared to 68% prescribed antipsychotics and 9% in receipt of HDAT in the baseline audit. The average cumulative dose for every category (oral medication, depot and both) was calculated with a range from 1% to 168% (mean = 70%) for oral antipsychotic (single/combined), 1% to 193% (mean = 50%) for depots and 20% to 257% (mean = 95%) for combination of oral and depot. This compares with ranges of 1.6% to 215% (mean = 44.3%) for oral antipsychotic (single/combined), 0.04% to 100% (mean = 25.8%) for depots and 21% to 425% (mean = 119.6%) for combination of oral and depot in the baseline audit. Similar to the baseline survey no patient met all seven audit criteria but there was better adherence overall with best practice guidance. Blood and ECG monitoring were the most consistent parameters measured.

**Conclusion:**

Lower HDAT was achieved post intervention. Results, whilst positive, indicate the need for ongoing audit to maintain best standards.

